# 1222. Ceftolozane-Tazobactam Heteroresistance in Cystic Fibrosis Related *Pseudomonas aeruginosa* Infections

**DOI:** 10.1093/ofid/ofab466.1414

**Published:** 2021-12-04

**Authors:** James Sanders, Marguerite Monogue, David E Greenberg, Christine A Pybus, Andrew E Clark

**Affiliations:** 1 University of Texas Southwestern Medical Center, Dallas, Texas; 2 University of Texas Southwestern, Dallas, TX; 3 UT SOUTHWESTERN MEDICAL CENTER, DALLAS, TX

## Abstract

**Background:**

Cystic fibrosis (CF) patients are often colonized with *Pseudomonas aeruginosa* (PSA). During treatment, PSA can develop subpopulations exhibiting variable *in vitro* antimicrobial susceptibility patterns. Heteroresistance may underlie the reported discordant *in vitro* results and clinical responses to various antimicrobials. Here, we sought to examine the presence and nature of PSA heteroresistance to ceftolozane-tazobactam (C-T) in isolates originating from CF pulmonary exacerbations.

**Methods:**

Respiratory cultures from 26 adult CF patients were collected. From each sample, 5-10 PSA colonies were selected. Susceptibility testing was conducted via E-test for C-T, ceftazidime-avibactam (CZA), and imipenem-relebactam (I-R). Polyclonal-heteroresistance (PHR) was defined as the presence of different susceptibility profiles among the colonies that originated from a single patient specimen. Population analysis profile (PAPs) were performed to assess the presence of monoclonal-heteroresistance (MHR), defined as ≥ 4 fold change in the C-T MIC from a single colony over 24-48 hours.

**Results:**

246 PSA isolates from 26 adult CF patients were included. The C-T MIC_50_ and MIC_90_ were 1/4 and ≥ 256/4 µg/mL, respectively (Figure 1). Sixteen of the 26 patients (62%) demonstrated ≥ 2 fold change in C-T MIC between isolates from the same culture. Of these 16 isolates, the fold change in C-T MIC was >2 fold for 7 isolates (27%) and resulted in a susceptibility interpretation change in 6 of the isolates (23%). Of the 32 isolates that underwent PAP testing, 7 grew on MH plates at 2-fold the C-T MIC concentration. One isolate, PSA 1311, demonstrated growth on PAPs up to 4 fold the MIC (16/4 µg/mL) (Figure 2).

Figure 1. Ceftolozane-tazobactam, ceftazidime-avibactam, and imipenem-relebactam MIC distributions against PSA isolates from 26 adult CF patients

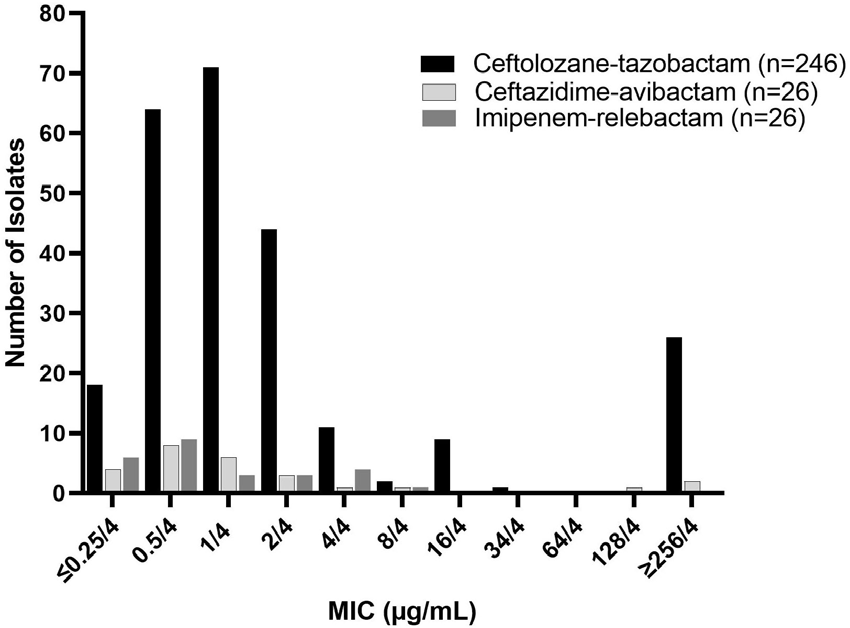

Figure 2. Monoclonal heteroresistance to C-T (PSA 1311 [C-T MIC 1/4 µg/mL] PAPs at 2, 4, 6, and 8-fold the C-T MIC).

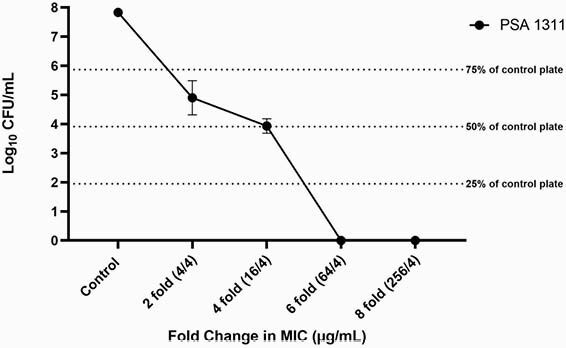

**Conclusion:**

Susceptibilities to C-T and CZA were similar across our CF PSA isolates. Comparatively, I-R retained better *in vitro* potency. C-T PHR exists among PSA isolates in the majority of our CF patients. Approximately 25% of these PHR isolates resulted in susceptibility interpretation changes supporting concerns surrounding the utility of traditional susceptibility testing methodology for CF isolates. These data suggest MHR also exists, albeit rare in this small subset. Additional data are needed to better understand these results in clinical context.

**Disclosures:**

**David E. Greenberg, MD**, **Shionogi** (Grant/Research Support)**Solenic Medical** (Shareholder)

